# Affordability and Access Challenges Among US Subscribers to Nongroup Insurance Plans

**DOI:** 10.1001/jamahealthforum.2021.5141

**Published:** 2022-02-18

**Authors:** Alison A. Galbraith, Joachim O. Hero, Alon Peltz, Jon Kingsdale, Rachel S. Gruver, Anna D. Sinaiko

**Affiliations:** 1 Department of Population Medicine, Harvard Pilgrim Health Care Institute, Harvard Medical School, Boston, Massachusetts; 2 Division of General Pediatrics, Boston Children’s Hospital, Boston, Massachusetts; 3 Now with RAND Corporation, Boston, Massachusetts; 4 Department of Health Law, Policy and Management, Boston University School of Public Health, Boston, Massachusetts; 5 Now with Department of Epidemiology, Columbia University Mailman School of Public Health, New York, New York; 6 Department of Health Policy and Management, Harvard T.H. Chan School of Public Health, Boston, Massachusetts

## Abstract

This cohort study assesses cost-related experiences in non-group plans purchased on or off Marketplace and variation by Marketplace enrollment, decision support use, and other characteristics.

## Introduction

The Affordable Care Act (ACA) expanded access to nongroup health insurance through insurance Marketplaces with subsidies and decision support. Health care affordability and accessibility in nongroup plans have improved, but challenges remain.^1,2,3,4,5^ This cohort study assessed cost-related experiences in nongroup plans purchased on or off the ACA Marketplace and variation by Marketplace enrollment, decision support use, and other characteristics.

## Methods

We conducted a panel survey linked to enrollment and claims data from a single insurer in Massachusetts, Maine, and New Hampshire. We surveyed a stratified random sample of subscribers aged 18 to 63 years in 2017 nongroup plans purchased on or off the ACA Marketplaces after open enrollment and again 1 year later. Predictors were enrollment on vs off Marketplace; household income; chronic conditions among family members sharing a plan; health insurance literacy; and use of brokers or navigators, cost estimators, or *provider finders* (tools used to search for in-network physicians, other health care professionals, and hospitals and other health care facilities) at enrollment. Outcomes included self-reported delayed or forgone care due to cost and financial burden, and high out-of-pocket health care costs (>10% of income) and high total spending (net premiums plus out-of-pocket costs >19.5% of income) from claims for all family members in the plan. Analyses excluded subscribers with 6 or fewer months of enrollment and were weighted for sampling design and subscriber nonresponse. We assessed the association between predictors and outcomes using multivariable logistic and linear regression models controlling for subscriber and family characteristics and including an interaction between Marketplace and income group and between Marketplace and family chronic conditions. We estimated the same models adding use of a broker or navigator, cost estimator, and provider finder tool as predictors. Analyses were conducted using Stata, version 14.2 (StataCorp LLC).

This study followed the American Association for Public Opinion Research (AAPOR) and Strengthening the Reporting of Observational Studies in Epidemiology (STROBE) reporting guidelines. The Harvard Pilgrim Institutional Review Board approved the study and waived the requirement for written informed consent. See eMethods in the Supplement for details.

## Results

Both surveys were completed by 1223 subscribers (response rate of 18%). Of the weighted sample of 1068 subscribers with more than 6 months of enrollment, 25% were aged 35 years or younger, 38% were aged 36 to 55 years, and 37% were aged 56 to 63 years; 47% were male. Of 1068 subscribers with more than 6 months of enrollment, 40.3% had delayed or forgone care due to cost, 41.1% had financial burden, 9.1% had high out-of-pocket costs, and 23.6% had high total spending. In off-Marketplace plans compared with in-Marketplace plans, those with incomes 250% or less of the federal poverty level (FPL) and 250% to 400% FPL had greater probability of high out-of-pocket costs and of high total spending; those with incomes 250% or less FPL also had higher mean total spending and greater probability of high total spending in off-Marketplace plans (Table 1). Families with chronic conditions had higher out-of-pocket spending than those without, but for families both with and without chronic conditions, mean total spending and probability of high total spending were greater in off-Marketplace than in-Marketplace plans. There were no differences in delayed/forgone care due to cost or financial burden based on Marketplace enrollment and few differences in outcomes by health insurance literacy or use of decision support (Table 2).

**Table 1.** Adjusted Estimates of Experiences for Enrollees in Nongroup Plans^a^

Characteristic	Delayed/forgone care (n = 841)^b^		Financial burden (n = 890)		High OOP costs (n = 595)^c^		High total spending (n = 595)^d^		Total spending (n = 595)	
	Predicted probability, %	*P* value	Predicted probability, %	*P* value	Predicted probability, %	*P* value	Predicted probability, %	*P* value	Predicted mean, $	*P* value
**Income**										
<250% FPL										
Marketplace	40.4	.45	43.9	.14	14.0	.002^e^	36.1	<.001^e^	6415	<.001^e^
Off Marketplace	34.2		56.6		43.9		72.3		11 036	
250%-400% FPL										
Marketplace	44.9	.46	45.9	.33	8.7	.92	15.6	<.001^e^	7576	<.001^e^
Off Marketplace	50.9		53.8		8.2		48.1		11 328	
>400% FPL										
Marketplace	40.9	.22	32.6	.46	1.4	.31	11.5	.33	11 158	.91
Off Marketplace	34.1		28.6		0.3		8.2		11 222	
**Family with chronic condition**										
Marketplace	41.7	.98	49.0	.92	11.8	.001^e^	27.6	<.001^e^	9386	<.001^e^
Off Marketplace	41.8		49.7		32.1		50.1		13 218	
**Family without chronic condition**										
Marketplace	41.5	.27	35.9	.11	5.0	.21	17.0	.001^e^	8072	<.001^e^
Off Marketplace	35.8		44.1		11.3		35.4		10 072	
Health insurance literacy tertile										
Lowest	45.4	.02	46.7	.02	7.8	.98	22.3	.95	8607	.20
Middle	40.5	.16	37.6	.70	9.6	.60	23.4	.86	8915	.52
Highest	34.0	NA	35.8	NA	7.9	NA	22.6	NA	9243	NA

Abbreviations: FPL, federal poverty level; NA, not applicable; OOP, out-of-pocket.

^a^ Results are predicted outcomes from logistic and linear models that were weighted using inverse probability weights to account for sampling and survey nonresponse and controlled for listed variables plus state, age, sex, race and ethnicity, education, employment, family size, having a child in the family, and interaction between Marketplace and income and between Marketplace and chronic condition.

^b^ Delayed/forgone care due to cost was measured only among enrollees who reported that they or a family member needed health care during 2017.

^c^ High OOP costs = OOP costs for health care services greater than 10% of income.

^d^ High total spending = OOP costs for health care services for all family members plus family premium (net of Advanced Premium Tax Credit) greater than 19.5% of family income.

^e^ Statistically significant based on Holm-Bonferroni correction.

**Table 2.** Adjusted Estimates of Experiences in Nongroup Plans Based on Use of Decision Support Tools and Resources During Plan Selection^a^

Decision support tool/resource use	Delayed/forgone care (n = 830)^b^		Financial burden (n = 879)		High OOP costs (n = 588)^c^		High total spending (n = 588)^d^		Total spending (n = 595)	
	Predicted probability, %	*P* value	Predicted probability, %	*P* value	Predicted probability, %	*P* value	Predicted probability, %	*P* value	Predicted mean, $	*P* value
**Used broker or navigator**										
Yes	37.5	.45	40.0	.82	3.6	.006^e^	24.0	.76	9060	.83
No, neither	41.3		41.1		9.1		22.6		8943	
**Used cost estimator**										
Yes	44.5	.08	44.2	.12	7.2	.44	21.5	.49	9067	.62
No	37.4		38.2		9.1		24.0		8880	
**Used provider finder**										
Yes	39.0	.48	41.8	.69	6.7	.22	24.8	.27	8967	.99
No	41.9		40.2		9.4		21.1		8962	

Abbreviation: OOP, out-of-pocket.

^a^ Adjusted results are from models that include all of the listed variables and Marketplace, income, chronic condition in family, health insurance literacy, state, age, sex, race and ethnicity, education, employment, family size, having a child in the family, and interaction between Marketplace and income and between Marketplace and chronic condition.

^b^ Delayed/forgone care due to cost was measured only among enrollees who reported that they or a family member needed health care during 2017.

^c^ High OOP costs = OOP costs for health care services greater than 10% of income.

^d^ High total spending = OOP costs for health care services for all family members plus family premium (net of Advanced Premium Tax Credit) greater than 19.5% of family income.

^e^ Statistically significant based on Holm-Bonferroni correction.

## Discussion

Affordability challenges were common among nongroup enrollees. For low-income, subsidy-eligible families, enrollment in Marketplace plans was associated with lower risk of high out-of-pocket and high total spending compared with enrollment off-Marketplace, although delayed/forgone care due to cost and financial burden did not differ. Use of brokers or navigators was associated with lower probability of high out-of-pocket costs, but otherwise experiences were no better with decision support. These results suggest that downstream cost-related experiences are less influenced by Marketplace shopping tools. Limitations of this study include low response rate, selection effects, a single regional carrier, a Massachusetts sample that did not include subsidy data or enrollees with incomes less than 300% FPL in a separate Marketplace program, and lack of Marketplace eligibility data. Despite lower out-of-pocket spending among subsidy-eligible Marketplace enrollees, negative cost-related experiences persist. Our findings suggest that building on ACA coverage gains by expanding eligibility and the amount of subsidies^6^ could address the remaining affordability challenges facing nongroup enrollees.

## Notes

Supplement.
eMethods.
