# Supplementary material for: Affordability and Access Challenges Among US Subscribers to Nongroup Insurance Plans
Source: JAMA Health Forum. 2022 Feb 18;3(2):e215141. doi: 10.1001/jamahealthforum.2021.5141 (PMC8903105; doi:10.1001/jamahealthforum.2021.5141)
Supplement: Supplement. — eMethods. [file jamahealthforum-e215141-s001.pdf]

Supplemental Online Content

Galbraith AA, Hero JO, Peltz A, Kingsdale J, Gruver RS, Sinaiko AD. Affordability and access challenges among US subscribers to nongroup insurance plans. *JAMA Health Forum*. 2022;3(2):e215141. doi:10.1001/jamahealthforum.2021.5141

eMethods.

This supplemental material has been provided by the authors to give readers additional information about their work.

© 2022 Galbraith AA et al. *JAMA Health Forum.*

eMethods

1) STUDY POPULATION

The study population included non-group health plan subscribers aged 18-63 years who purchased coverage on or off the ACA Marketplace from a large non-profit regional insurance carrier during 2017 open enrollment in Massachusetts, New Hampshire, or Maine. Subscribers were identified by the insurance carrier as the person purchasing the coverage; they were not necessarily the head of the household or the oldest person in the household. We identified 25,621 subscribers and selected a random sample of 1,300 subscribers from each of six strata based on state and enrollment source (on vs. off-Marketplace); we selected all off-Marketplace subscribers in Maine as only 706 were available. A total of 7,206 subscribers were selected.

New Hampshire and Maine use the federal HealthCare.gov Marketplace, while Massachusetts has a state-based Marketplace. Our study population does not include subscribers in the separate Connector Care Marketplace program in Massachusetts for those with household incomes below 300% of the federal poverty level (FPL) because the insurance carrier for our study does not participate in this program.

2) SURVEY DATA

a. <u>Baseline survey</u>

A total of 7,206 subscribers were mailed a baseline survey after 2017 Open Enrollment. The baseline survey was completed by 2,029 subscribers (American Association for Public Opinion Research (AAPOR) response rate type 2 of 29%). The survey collected information about the subscriber and any family members sharing their plan. Respondents received a $20 gift card incentive after completion of the survey. Additional baseline survey details have been published previously.[1]

The baseline survey collected demographic and other <u>data on the subscriber</u> as the person responsible for decisions about plan choice for the family. These included the subscriber's highest level of education, race and ethnicity, employment, and health insurance coverage type in 2016. We measured self-reported race and ethnicity in the survey because race and ethnicity have been shown to be associated with health care use and access to care in prior studies. To measure subscriber race and ethnicity, the survey asked subscribers to report if they were Hispanic or not, and asked subscribers to indicate if their race was one or more of the following: American Indian or Alaska Native, Asian, Black or African-American, Native Hawaiian or other Pacific Islander, White, or Other. These measures were combined to create a mutually exclusive binary variable for whether the subscriber was Non-Hispanic White or not, with those selecting more than one race categorized as not Non-Hispanic White. The survey measured subscriber health insurance literacy, using 13 questions that assessed confidence with understanding insurance terms and performing insurance-related actions such as finding a covered provider.[2] Responses to the health insurance literacy questions were aggregated and categorized into tertiles within the sample. Subscribers were also asked if they used a broker or navigator, cost estimator, or provider finder tool during plan selection.

The baseline survey also collected <u>data on the subscriber's family</u>. Subscribers were asked about the presence of chronic conditions among members of the subscriber's family that shared the health plan, based on the following survey item:

> A chronic condition is a health condition that has lasted or is expected to last a year or longer, may limit what one can do, and may require ongoing care, such as diabetes, heart disease, or asthma. Do you or any of your family members covered by your health plan have a chronic condition?
> - ☐ Yes
> - ☐ No

b. <u>Follow-up survey</u>

Of the 2,029 subscribers completing the baseline survey, 92% (1,859) gave permission to be contacted again. One year later, we sent a follow-up survey via mail or email to these 1,859 subscribers, regardless of whether they remained enrolled in a non-group plan with the carrier. Respondents received a $20 gift card incentive after completion of the survey. Follow-up surveys were completed by 1,223 subscribers, (AAPOR response rate type 2 of 18% of the population initially sampled, and 60% of those who completed the baseline survey). Of these, we excluded 155 (12.7%) who had stayed in the 2017 plan for six months or less, yielding a follow-up survey study population of 1,068 subscribers along with their families.

The follow-up survey collected data on household income in 2017, which was measured with the following question:

> Income can affect the plans and subsidies for which you are eligible. In 2017, approximately what was your family's total income from all sources, before taxes?
> - ☐ Less than $10,000
> - ☐ $10,001 to $20,000
> - ☐ $20,001 to $25,000
> - ☐ $25,001 to $30,000
> - ☐ $30,001 to $35,000
> - ☐ $35,001 to $40,000
> - ☐ $40,001 to $50,000
> - ☐ $50,001 to $60,000
> - ☐ $60,001 to $75,000
> - ☐ $75,001 to $100,000
> - ☐ $100,001 to $125,000
> - ☐ $125,001 or more

Family income was assigned as the midpoint between the chosen income category thresholds for all categories except for the highest. For incomes over $125,000, we used the median income over 125,000 in each state by self-reported household size from the 5-year American Community Survey (2013-2017). Missing values were assigned their 2016 income reported in the baseline survey if available.

The follow-up survey also asked about household size; this was used together with family income to estimate family income as a percent of the FPL.

3) ADMINISTRATIVE DATA

a. Enrollment data

Enrollment data on subscriber demographics, family members in the plan, and 2017 plan attributes were obtained from the insurance carrier and linked to survey data at the subscriber level. Administrative variables included state, coverage source (on vs. off-Marketplace), enrollment duration, plan metal tier, CSR designation, subscriber age and sex, number and ages of family members in the plan, monthly premium, and advance premium tax credit (APTC) subsidy amounts for each family contract. APTC data were not available for Massachusetts study subjects, most of whom were unlikely to have had large APTCs given that those with incomes <300% FPL who were eligible for more substantial APTCs would likely have enrolled through the separate Connector Care program in which the carrier for our study does not participate. Using geocoded subscriber address data, we obtained subscriber census block socioeconomic data to describe the study sample relative to the larger population from which it was drawn and to weight analyses for non-response.

b. Claims data

For the 62% (759) of the 1,223 follow-up survey respondents who gave permission, we obtained medical and pharmacy claims data on 2017 out-of-pocket (OOP) health care spending for all family members enrolled on the plan, which were used to calculate family-level OOP costs and total spending, as described

© 2022 Galbraith AA et al. *JAMA Health Forum.*

below. Of the 759 families with claims data available, 673 were enrolled for more than 6 months and were included in analyses of OOP costs and total spending.

4) OUTCOMES: MEASUREMENT OF NEGATIVE PLAN EXPERIENCES

a. From follow-up survey data

Delayed/forgone care due to cost

Subscribers responding yes to the survey question below were considered to have delayed/forgone care due to cost.

In 2017, was there any time when you or a family member on your plan delayed or didn't get a doctor visit, test, prescription, or any other medication care because of the cost?

- ☐ Yes
- ☐ No
- ☐ No one needed any medical services in 2017

Those responding "no one needed any medical services in 2017" were excluded from the denominator for this measure.

Financial burden

Subscribers responding yes to any of the three questions below were considered to have financial burden.

In 2017, was there any time when you had trouble paying or were unable to afford any of your or your family's medical bills?

- ☐ Yes
- ☐ No

In 2017, did you have to set up a payment plan with a hospital of doctor's office?

- ☐ Yes
- ☐ No

In 2017, did you have trouble paying for other basic bills like food, heat, or rent because of medical costs for you or a member of your family?

- ☐ Yes
- ☐ No

b. From administrative and claims data

High OOP costs

High OOP costs were defined as OOP costs for health care services for all family members that exceeded 10% of family income.[3-5] We aggregated OOP health care costs from medical and pharmacy claims for each family member in the subscriber's plan in 2017 and annualized them from the number of months the family was enrolled in 2017. Family income (measured from follow-up survey data as described above) was used as the denominator to calculate 2017 OOP costs as a percent of income, based on methods used in other studies.

Total spending

© 2022 Galbraith AA et al. *JAMA Health Forum.*

We calculated 2017 total spending as the sum of all family members' OOP health care costs plus premiums after subsidy. Annualized OOP costs for health services and family income were defined as described above. Premium spending was calculated from monthly premium rates obtained from the carrier, which were annualized based on the number of months enrolled in 2017.

High total spending

High total spending was defined as annual OOP costs for health care services and premiums for all family members that exceeded 19.5% of family income. This threshold combines a 10% threshold for OOP costs with the 9.5% threshold used in the ACA to define affordable premiums for employer-sponsored coverage.[5,6] Family income (measured from follow-up survey as described above) was used as the denominator to calculate 2017 total spending as a percent of income.

5) SAMPLING AND NON-RESPONSE WEIGHTS

All analyses were weighted to account for elements of our sampling design and for non-response to the baseline and follow-up surveys. Because subscribers were sampled equally from state-enrollment source strata (except for enrollees off-Marketplace in Maine, where fewer enrollees were available), some subscribers had a higher probability of being included in the sample than others. Sample weights were created to adjust our sample back to the population from which it was drawn (all non-group market subscribers from the carrier in Massachusetts, New Hampshire, and Maine). To create weights for non-response to the baseline survey, we used administrative data to model the likelihood of response (vs. non-response) to calculate inverse probability weights. We followed steps as suggested in Seaman & White (2013)[7] which entails using forward selection based upon AIC to add predictors, including interaction terms, to identify a non-response model. Model fit for the non-response model was tested using Hosmer-Lemeshow and Le Cassie-van Houwelingen methods,[8] which did not detect evidence of poor model fit, and weights were examined for extreme values, of which none were discovered. The final baseline survey non-response model reflected Marketplace enrollment, state, sex, age, metal tier of subscriber health plan, percent of census block that is Hispanic, whether the enrollee chose a new plan in 2017, and several interaction terms.

We used a similar process to calculate weights for non-response to the follow-up survey but added baseline survey data as well as administrative data to model the likelihood of response (vs. non-response) to the follow-up survey among baseline respondents to calculate inverse probability weights. The final follow-up survey non-response model reflected Marketplace participation, state, sex, nativity, rating of experience trying to choose a plan, percent of census block that is low socioeconomic status, and the interaction of state and Marketplace participation. To create the final weights used in analyses of the follow-up survey, we multiplied the original weight from the baseline survey by the weight that reflects the likelihood of responding to the follow-up survey. Characteristics of the final weighted sample, unweighted sample, and full population from which they were drawn (i.e. all non-group subscribers enrolled with the carrier in 2017) can be found in eTable 1.

6) ANALYSIS

All analyses were weighted using inverse probability weights to account for sampling design and subject-level nonresponse. Data were analyzed at the family level, where single subscribers were considered a family of one. We limited analyses to families whose plan experiences and health care spending were based upon enrollment of more than six months in 2017.

We assessed whether Marketplace enrollment, income, having a chronic condition in the family, and health insurance literacy were associated with binary outcomes using multivariable logistic regression models. We assessed whether these characteristics were associated with total spending using linear regression models. We use this as our main specification for the total spending model because prior analyses find that linear models predict health care spending similarly to models with more complex functional forms.[9]

eTable 1. Characteristics of Subscribers with Non-Group Plans in the Study Population and in the Larger Population from the Carrier in 2017

© 2022 Galbraith AA et al. *JAMA Health Forum.*

	Total study population (unweighted) No. (%)	Total study population (weighted) %	All subscribers in non-group plans from the carrier in 2017 (unweighted) No. (%)
**Total**	n=1068	n=1,068	n=25,621
State			
ME	346 (32)	44%	12,266 (48)
NH	435 (41)	33%	8,095 (32)
MA	287 (27)	22%	5,260 (21)
**Subscriber characteristics**			
Age (years)			
≤35	200 (19)	25%	6,112 (24)
36-45	135 (13)	15%	4,501 (18)
46-55	251 (24)	23%	6,570 (26)
56-63	482 (45)	37%	8,438 (33)
Sex			
Female	637 (60)		13,755 (54)
Male	431 (40)	47%	11,866 (46)
Enrolled as an individual	653 (61)	66%	16,632 (65)
**Family and plan characteristics**			
Plan includes a child < 18 years	172 (16)	15%	3,898 (15)
Received APTC	323 (30)	48%	12,918 (50)
CSR plan	162 (15)	26%	6,609 (26)
Metal tier			
Bronze	435 (41)	37%	9,738 (38)
Silver	430 (40)	46%	12,129 (47)
Gold	156 (15)	13%	2,881 (11)
Platinum	47 (4)	4%	873 (3)
**Neighborhood variables**			
Residence in census tract with >20% of residents below poverty	24 (2)	3%	937 (4)
Residence in census tract with >25% without high school degree	9 (1)	2%	625 (2)
Percent Black in census tract (mean %, SD)	1 (4)	1%	1 (5)
Percent Hispanic in census tract (mean %, SD)	2 (3)	2%	2 (5)

All n's are unweighted. Percentages may not add up to 100% due to rounding. ME = Maine. NH = New Hampshire. MA = Massachusetts. APTC = advanced premium tax credit. CSR = cost-sharing reduction.

Models included covariates selected a priori and included subscriber covariates (age, sex, education, race/ethnicity, employment, health insurance coverage type in 2016) and family covariates (family size, having a child < 18 years old in the plan, state). Because the association between Marketplace enrollment and outcomes could depend on whether enrollees were eligible for subsidies, the models included an interaction term between Marketplace and income group. We also added an interaction term between Marketplace and presence of a chronic conditions in the family to assess whether the association between having a chronic condition in the family and negative cost-related experiences varied on vs. off Marketplace.

We evaluated whether particular decision support tools used during plan selection were associated with negative plan experiences. We estimated the same models as above with the addition of variables measuring self-reported use of a broker or navigator, of a cost estimator, and of a provider finder tool.

© 2022 Galbraith AA et al. *JAMA Health Forum.*

All results are reported as predicted probabilities or predicted means from these models, and adjusted for multiple comparisons using the Holm-Bonferroni correction.[10]

7) REFERENCES

1. Hero JO, Sinaiko AD, Kingsdale J, Gruver RS, Galbraith AA. Decision-making experiences of consumers choosing individual-market health insurance plans. *Health Aff.* 2019;38(3):464-472.
2. Holahan J, Long SK. Health reform monitoring survey, United States, first quarter 2016. Ann Arbor, MI: Inter-university consortium for political and social research (ICPSR 36744). https://doi.org/10.3886/ICPSR36744.v2. Accessed Sep 17, 2019.
3. Liu C, Gotanda H, Khullar D, Rice T, Tsugawa Y. The Affordable Care Act's insurance marketplace subsidies were associated with reduced financial burden for US adults. *Health Aff.* 2021;40(3):496-504.
4. Wisk LE, Peltz A, Galbraith AA. Changes in health care–related financial burden for US families with children associated with the Affordable Care Act. *JAMA Pediatrics.* 2020;174(11):1032-1040.
5. Goldman AL, Woolhandler S, Himmelstein DU, Bor DH, McCormick D. Out-of-pocket spending and premium contributions after implementation of the Affordable Care Act. *JAMA Intern Med.* 2018;178(3):347-355.
6. Liu C, Tsugawa Y, Weiser TG, Scott JW, Spain DA, Maggard-Gibbons M. Association of the US Affordable Care Act with out-of-pocket spending and catastrophic health expenditures among adult patients with traumatic injury. *JAMA Netw Open.* 2020;3(2):e200157-e200157.
7. Seaman SR, White IR. Review of inverse probability weighting for dealing with missing data. *Stat Methods Med Res.* 2013;22(3):278-295.
8. Hosmer DW, Lemeshow S, Sturdivant RX. *Applied Logistic Regression.* John Wiley & Sons, Inc.; 2013.
9. Buntin MB, Zaslavsky AM. Too much ado about two-part models and transformation?: Comparing methods of modeling Medicare expenditures. *J Health Econ.* 2004;23(3):525-542.
10. Holm S. A Simple Sequentially Rejective Multiple Test Procedure. *Scandinavian Journal of Statistics.* 1979;6(2):65-70.

© 2022 Galbraith AA et al. *JAMA Health Forum.*
